# Molecular study of the status of *Angiostrongylus cantonensis* in rats in Haiti

**DOI:** 10.1051/parasite/2024063

**Published:** 2024-10-09

**Authors:** Jimmy Fedna, Romain Borne, Dominique Rieffel, Gudrun Bornette, Jean-Hugues Henrys, Frédéric Grenouillet, Francis Raoul

**Affiliations:** 1 Université de Franche-Comté, CNRS, Chrono-environnement 25000 Besançon France; 2 Université de Franche-Comté, CHU Besançon, CNRS, Chrono-environnement 25000 Besançon France; 3 Équipe de Recherche sur l’Écologie des Maladies Infectieuses et Tropicales (EREMIT) 6110 Port-au-Prince Haïti

**Keywords:** *Angiostrongylus cantonensis*, *Eosinophilic meningitis*, Rats, One Health, Haiti

## Abstract

*Angiostrongylus cantonensis*, commonly known as the rat lungworm, causes Eosinophilic meningitis in humans. Our study aimed to investigate the prevalence and distribution of this parasite in rats in Haiti. Rats were trapped at 8 sites, 7 in Artibonite (rural region) and one in an urban area of Port-au-Prince. After euthanasia, hearts and lungs were sampled and preserved in 70% ethanol. Subsequently, the organs were dissected to detect adult worms. Parasite DNA was amplified using PCR targeting either the nematode ITS2 gene for rodent lung tissue or *cox1* for isolated worms. Subsequent sequencing allowed parasite identification. A total of 70 rats were captured, *i.e.* 23 *Rattus norvegicus* and 47 *Rattus rattus.* Adult nematodes morphologically compatible with *A. cantonensis* were isolated from 5/70 rats (7%) and identification was confirmed by sequencing. Molecular analysis of lung tissue revealed a parasite prevalence of 31.4% (22/70), and its presence at 4 of the 8 sites investigated, including Port-au-Prince. The molecular approach on lung tissue targeting the ITS2 gene enabled us to detect a prevalence 4 times higher than the visual search for adult worms alone. Only one COX1 haplotype was identified, belonging to genotype II-G, widely distributed in Brazil, the French Antilles (Guadeloupe), French Polynesia, Hawaii, and Japan. These results confirm that *A. cantonensis* is an endemic parasite in Haiti not only in the capital Port-au-Prince, but also in several rural areas. Direct molecular screening for *Angiostrongylus* DNA in rat lung tissue showed higher sensitivity than visual detection of worms during dissection and could be useful for further prevalence studies.

## Abstract (fr)

*Angiostrongylus cantonensis*, communément appelé ver pulmonaire du rat, provoque la méningite éosinophile chez l’homme. Notre étude visait à étudier la prévalence et la distribution de ce parasite chez le rat en Haïti. Les rats ont été piégés dans 8 sites, 7 dans l’Artibonite (région rurale) et un dans une zone urbaine de Port-au-Prince. Après euthanasie, les cœurs et les poumons ont été échantillonnés et conservés dans de l’éthanol à 70 %. Par la suite, les organes ont été disséqués pour détecter les vers adultes. L’ADN du parasite a été amplifié par PCR ciblant soit le gène ITS2 des nématodes pour le tissu pulmonaire des rongeurs, soit *cox1* pour les vers isolés. Le séquençage ultérieur a permis l’identification du parasite. Soixante-dix rats ont été capturés, soit 23 *Rattus norvegicus* et 47 *Rattus rattus*. Des nématodes adultes morphologiquement compatibles avec *A. cantonensis* ont été isolés chez 5/70 rats (7 %) et l’identification a été confirmée par séquençage. L’analyse moléculaire du tissu pulmonaire a révélé une prévalence du parasite de 31,4 % (22/70), et sa présence dans 4 des 8 sites étudiés, dont Port-au-Prince. L’approche moléculaire sur les poumons ciblant le gène ITS2 nous a permis de détecter une prévalence 4 fois supérieure à la recherche visuelle des seuls vers adultes. Un seul haplotype *cox1* a été identifié, appartenant au génotype II-G, largement distribué au Brésil, aux Antilles françaises (Guadeloupe), en Polynésie française, à Hawaï et au Japon. Ces résultats confirment qu’*A. cantonensis* est un parasite endémique en Haïti non seulement dans la capitale Port-au-Prince, mais aussi dans plusieurs zones rurales. Le dépistage moléculaire direct de l’ADN d’*Angiostrongylus* dans le tissu pulmonaire du rat a montré une sensibilité supérieure à la détection visuelle des vers lors de la dissection et pourrait être utile pour d’autres études de prévalence.

## Abstract (ht)

*Angiostrongylus cantonensis* se yon vè parazit nematòd yo konn rele ann anglè « rat lungworm » . Sa vle di se yon vè nou jwenn nan poumon rat. Vè sa a ka lakòz menenjit eozinofil kay moun. Objektif rechèch nou se te envestige prevalans ak distribisyon parazit sa a pami rat ann Ayiti. Nou te kaptire rat yo nan 8 sit diferan. Sèt sit nan Latibonit (zòn riral) ak youn nan nan Pòtoprens (zòn iben). Apre nou fin touye rat yo, nou te pran kè ak poumon yo epi n te mete nan etanòl (konsantrasyon 70%) pou nou te konsève yo. Apre sa, nou te diseke ògan yo pou n detekte vè parazit la. Nou te ekstrè ADN parazit la epi nou te sèvi ak apwòch molekilè PCR pou n anplifye ADN lan epi n te sible jèn nikleyè ITS2 nan ti moso poumon rat yo oswa *cox1* nan vè nou te rive retire nan ògan rat yo. Aprè sa, nou te sekanse jèn sa yo pou n te ka idantifye parazit la kòm sa dwa. Nou te rive jwenn yon total 70 rat. Pami yo, te gen 23 espès rat *Rattus norvegicus* ak 47 espès rat *Rattus rattus*. Nou te retire vè adilt parazit la lakay 5 rat (5 rat sou 70 egal 7%). Fòm vè yo ak anpil lòt karakteristik yo te sanble tèt koupe ak *A. cantonensis* ki se vè n ap chachè a epi rezilta sekansaj yo te konfime sa. Nan kè yon rat, nou te retire yon vè *A. cantonensis*; men, PCR la pa t rive konfime sa. Analiz PCR sou moso poumon yo te revele yon prevalans parazit la (31,4% egal 22/70), ak prezans li nan 4 pami 8 sit nou te envestige yo — sit Pòtoprens lan ladann tou. Apwòch molekilè sou poumon yo te pèmèt nou mezire yon prevalans 4 fwa pi wo pase lè n ap chachè vè adilt yo ak mikwoskòp sèlman. Nou te idantifye yon sèl aplotip COX1 ann Ayiti. *cox1* fè pati jenotip klad II-G ki se klad ki simaye pi plis sou latè. Yo jwenn li nan peyi Brezil, Gwadloup, Polinezi franse, Awayi ak Japon. Rezilta sa yo konfime *A. cantonensis* se yon parazit ki andemik ann Ayiti; li blayi kò li kote ki pi lwen pase Pòtoprens. Sa mete aksan sou risk sante pou popilasyon an. Se tès molekilè dirèk nan poumon rat ki te montre pi wo nivo sansiblite; egal : tès sa a ka sèvi pou pwochen rechèch sou prevalans.

## Introduction

*Angiostrongylus cantonensis* (Chen 1935), the rat lungworm, is a nematode (phylum Nematoda) in the superfamily Metastrongyloidea and family Angiostrongylidae [2, 43]. This parasite is the most common cause of eosinophilic meningitis in humans [4]. Its lifecycle includes gastropods (snails or slugs) as intermediate hosts and rats as definitive hosts. The parasite has low host specificity: several rodent species (at least 17 according to Ref. [4]) and a wide variety of gastropods (at least 199 species) can be infected as definitive and intermediate hosts, respectively, and various species of vertebrates and invertebrates can act as paratenic hosts [4, 18]. Humans become infected mainly by consuming uncooked or poorly cooked gastropods or paratenic hosts (e.g. crustaceans, lizards, frogs, and toads) containing L3 larvae, which are the infectious stage of the parasite [42].

The parasite is endemic in equatorial and tropical climates, where more than 30 countries have reported human cases of neuroangiostrongylosis [45]. Most cases of *A. cantonensis*-associated eosinophilic meningitis occur in Southeast Asia, including China, and the Pacific Islands, where Hawaii could be considered a hotspot for the disease. There have been numerous cases in Europe and elsewhere of travellers returning from vacation in tropical/subtropical parts of the world [3, 11]. There was one case in France, with no known source of infection but without travel history abroad within the two years preceding diagnosis, suggesting human contamination outside the historical endemic area [30]. The frequency of reports regarding *A. cantonensis* in various hosts is increasing, particularly in subtropical and even temperate regions, with the first instance in continental Europe (Valencia, Spain) [13, 32], in South America as far south as Buenos Aires [19], and in North America as far north as Atlanta, Georgia [17]. Such recent range expansions may be a result of global warming, the diversity of potential intermediate hosts in these regions, and the ongoing efficient dispersal of infected rats carried by ships [4, 9, 14].

Among the Caribbean islands, the parasite has been reported in rats and/or gastropods in Cuba, the Dominican Republic, Grenada, Haiti, Guadeloupe, Puerto Rico, and Jamaica [1, 5, 6, 14, 35, 44, 46]. Raccurt *et al.* noted for the first time the presence of *A. cantonensis* in Haiti in 2002 [35]. They found the parasite in 30% of rats captured (7/23) in Port-au-Prince in two neighbourhoods (Damien and Carrefour) between March and July 2002. According to these researchers, in Haiti, 1.3% of the human deaths recorded in 1999 were due to meningitis of unknown aetiology [35].

Two decades later, a broader assessment of angiostrongylosis in Haiti was still needed. We therefore set up a survey to update the prevalence of *A. cantonensis* in definitive hosts in Haiti. Our investigation focused on the historical site of identification of the worm, *i.e.* Damien in the Port-au-Prince area, and included a new area, the Artibonite rural department. The Artibonite department is a major producer of cereals and foodstuffs on the island, and is characterised by its many rice paddies and wetlands. In the context of the global spread of the worm as a possible result of global changes [7], the present study therefore aimed to update information about the status of *A. cantonensis* in Haiti, and to improve the knowledge of its distribution.

## Materials and methods

### Ethics statement

Our samples did not include endangered or protected species. The protocol was validated by the Ethics Committee of the State University of Haiti, and a professor of veterinary medicine and an anaesthesiologist supervised our work in the field.

The rodents were not abused and they were manipulated and humanly killed in accordance with a European Directive (https://eur-lex.europa.eu/LexUriServ/LexUriServ.do?uri=OJ:L:2010:276:0033:0079:fr:PDF). Protocols were based on guidelines from Sikes *et al.* [39].

### Study area

**Figure 1:**
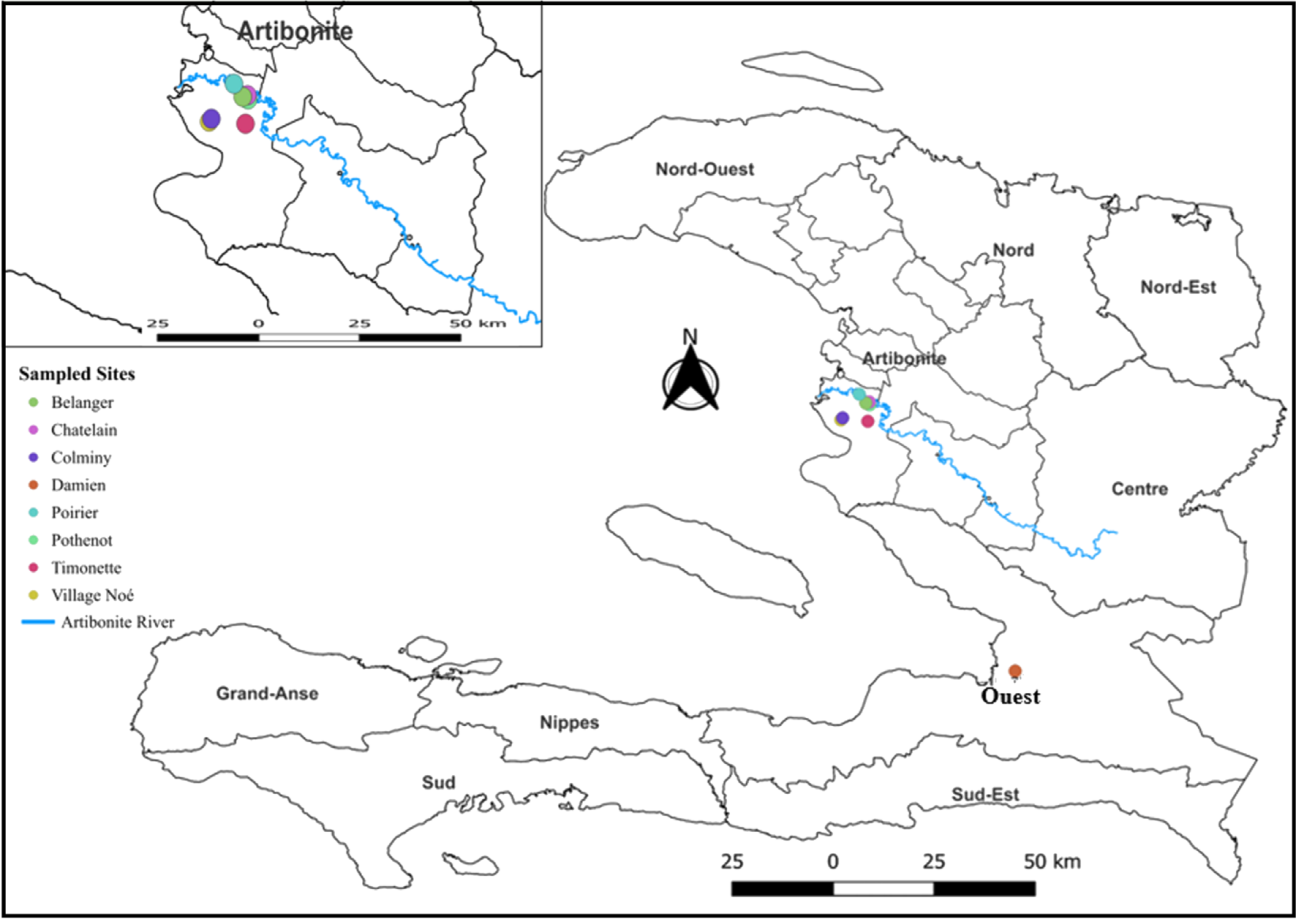
Map of Haiti with the departments, sampled sites.

The Republic of Haiti is a Caribbean country that occupies the western part of the island of Hispaniola, sharing the island with the Dominican Republic. The study was conducted in the rural area of Bocozelle, 5th communal section of the commune of Saint-Marc in the department of Artibonite, and in Damien, the urban area of Port-au-Prince. The department of Artibonite is the second most populous department of Haiti (estimated population was 1,727,5241 inhabitants [23], after that of Port-au-Prince (Western department) (Fig. 1) and is the breadbasket of the country, especially regarding rice production.

Seven sites were chosen in Bocozelle (Belanger, Chatelain, Colminy, Pothenot, Poirier, Timonette, and Village Noé) taking into account the diversity of habitats (rice fields, dry crops such as onions), and one site was sampled in Damien, Port-au-Prince, previously sampled by Raccurt *et al.* [35]. The Artibonite and Western departments share the same climate: tropical with two distinct seasons, a dry season from November to March and a rainy season from April to October.

### Rodent capture

The entire sampling procedure was carried out following protocols described in “Protocols for field and laboratory rodent studies” [21]. In November 2020 (end of the rainy season) and September 2021 (rainy season), we captured rats using live traps made locally (36 × 30 × 20 cm). These traps were baited with rice, herring, salami, and “Chico”, a traditional Haitian cheese puff snack (known as a very attractive food for rats, according to local people).

At each site, we set up trap lines of 10–15 traps, with traps spaced 5–10 m apart. The traps were set at dusk and checked at dawn. Traps containing rats were removed and immediately replaced with new traps. The number of traps and the number of days spent at a site varied according to logistical constraints and the country’s socio-political unrest. The capture success rate was determined by the following formula:

Number of rats captured on site / Total number of trapping attempts (i.e. number of trapping nights * number of traps set) × 100.

### Dissection and morphological identification of rats

A field laboratory was set up. Rats were anaesthetised with isoflurane, euthanised by cervical dislocation, and then dissected. They were weighed and sexed. Removed organs (hearts, lungs) were preserved in 70% ethanol and stored to be examined later for adult worm detection. The determination of each rodent age class was based on the crystalline lens weight method [20, 28, 40]. Crystalline lenses were stored in 10% formalin. To our knowledge, there are only 2 species of rats in Haiti (Jacques Blaise, personal communication): the brown rat *R. norvegicus* and the black rat *R. rattus*. Using the equations of Hardy *et al.* [20] for *R. norvegicus* and those of Tanikawa [40] for *R. rattus*, the rats were divided into age groups (in months) according to their crystalline lens weight.

### Worm collection

The heart and lungs collected were dissected and carefully examined under a stereomicroscope to collect the parasites. Adult worms were manually extracted with fine-tipped forceps from the heart, pulmonary trunk, and ramifications of pulmonary arteries in the lungs. The worms were clarified in lactophenol and identified using an optical microscope, based on male caudal bursa morphology and spicule size, which are generally used as taxonomic characteristics for *Angiostrongylus* species identification [26, 27].

### Molecular identification

#### Strategy

Firstly, molecular analysis of the adult worms taken from the rats was carried out for species identification. For this, we extracted the DNA from each worm, then performed PCR amplification targeting the *cox1* gene, followed by sequencing of the amplified DNA [13, 36].

Secondly, we looked for the presence of parasites in the organs taken from the rats. To do this, we performed pan-strongylid PCR targeting the ITS2 gene [16] directly on lung DNA extracts from all rats, followed by sequencing, to determine the molecular prevalence of *A. cantonensis* and to assess potential infection or coinfection with other strongylid worms. This ITS2 region is widely used to differentiate strongylid nematode species since its sequence size can vary within species [15, 37].

Finally, the identification of *Rattus* species (*R. norvegicus* or *R. rattus*) was done by restriction fragment length polymorphism (RFLP) analysis on their mitochondrial *cytb* gene. Based on the work of Galan *et al.* [12] who targeted a small region of the mitochondrial *cytb* gene (130 bp) sufficient for rodent species identification using next-generation sequencing, we designed primers to amplify a larger region (643 bp) in the *cytb* gene in order to perform RFLP analysis on this fragment to visually identify rodent species directly on gels, without any sequencing required [29, 33]. To validate this approach, we performed *in silico* PCR using Primer Blast (NCBI) and we obtained 153 different species (birds, mammals, including rodents). We investigated these sequences to determine the most relevant restriction enzyme with the online tool Restriction Enzyme Digest (http://insilico.ehu.es/restriction/main/) [38]: the *Rsa*I enzyme appeared to provide the most discriminant profiles between many genera, especially for the genus *Rattus*.

#### DNA extraction

DNA was extracted from 25 mg of tissue using a QIAamp Fast DNA Tissue Kit according to the manufacturer’s instructions (QIAGEN, Hilden, Germany), with the following optimisations. Storage ethanol desorption was performed placing each sample in 1 mL of 90%, 60%, 30% ethanol solution, and purified water for 1 h in turn. Then, mechanical and thermal lysis was performed by shaking for 10 min using a vortex and then incubation in a thermomixer for 1 h 30 min at 56 °C and 1 000 rpm. DNA concentrations were measured on a Quantus fluorimeter using a QuantiFluor One DNA kit (Promega, Madison, WI, USA).

#### PCR amplification and sequencing

Primers used in this study are listed in Table 1. For PCR amplification, 5 μL of extracted DNA matrix was added to 20 μL of a mixture of 1× EmeraldAmp GT PCR Master Mix (Takara Bio, Shiga, Japan), and 0.4 μM of each primer. PCR amplification was carried out using a thermocycler applying initial denaturation at 94 °C for 2 min, then 40 cycles of 94 °C for 1 min, 55 °C for 1 min, and 72 °C for 1 min, with a final elongation at 72 °C for 5 min. Aliquots (2 μL) of each PCR product were analysed by electrophoresis on a TAE 1×-agarose 1% gel containing SYBR Safe stain (Invitrogen, Waltham, MA, USA), applying a 6 V cm^−1^ electric field, and visualised using a Gel Doc XR+ system (Bio-Rad, Hercules, CA, USA) controlled by Image Lab Software.

**Table 1:** Primers used in this study.

	Sequences	Target	Purpose	Ref.
Pan-strongylid				
PCR				
NC1	5′–ACGTCTGGTTCAGGGTTGTT–3′	nuclear ITS2 (nematodes)	Identification of *A. cantonensis* in rodent organs	This study [15, 16]
NC2	5′–TTAGTTTCTTTTCCTCCGCT–3′			
*Sequencing*	5′–GTGCGTTTTGTGCGTTTAC–3′			
NC internal				
Worm identification				
AngiCOI_forward	5′–TTTTTTGGGCATCCTGAGGTTTAT–3′	*cox1* (*Angiostrongylus* spp.)	Taxonomic assessment of isolated worms	[13, 36]
AngiCOI_reverse	5′–CGAGGATAACCATGTAAACCAGC–3′			
*Rattus* identification				
For-cytB-Rattus	5′–CTCCCATGAGGACAAATATC–3′	cytb (rodents)	Confirmation of rodent species by RFLP	This study
Rev-cytB-Rattus	5′–ATGGGTGTTCTACTGGTTG–3′			

Amplicons were Sanger sequenced by Genewiz Genomics Service (Genewiz-Azenta, Leipzig, Germany) using a GA3730 DNA Analyser (Applied Biosystems, Waltham, MA, USA). Primers used for PCR amplification were used for sequencing. In the case of multiple signals on the sequencing chromatogram for ITS2, suggesting the presence of several nematode species in the amplicon, the newly designed NC-internal primer, which was designed to be highly specific to *A. cantonensis*, was used for a second sequencing run (Table 1). Sequences were identified by BLASTn analysis, using the NCBI GenBank database.

### Restriction fragment length polymorphism-PCR

After amplification of the *cytb* mitochondrial gene of rats, digestion of amplicons was performed using FastDigest *Rsa*I. Species identification of rats was based on the following restriction profiles: 188/455 bp for *R. rattus* and 144/188/311 bp for *R. norvegicus*.

### Phylogenetic analysis

A phylogenetic analysis was performed to compare the DNA sequences of *cox1* from adult worms in our study with other sequences available in GenBank. Sequences were aligned with the multiple alignment using fast Fourier transform (MAFFT) method [24] and cleaned following the block mapping and gathering with entropy (BMGE) approach [8]; then phylogenetic analysis was performed with Mr Bayes [22] in the workflow from the NGPhylogeny.fr online tool [25]. Phylogenetic tree visualisation was performed with the iTOL tool (https://itol.embl.de/tree/).

### Data analysis

R version 4.2.2 (2022-10-31 ucrt) was used to perform the analyses. Prevalence was defined in our study as: (number of infected rats/numbers of examined rats *100. Chi-squared or Fisher exact tests were performed to examine the association between prevalence of *A. cantonensis* and/or other nematodes and rat species.

## Results

### Sampling of rats

In total, 70 rats were captured at 7 of the 8 investigated sites. Capture success rates were 11% at Damien (Port-au-Prince), 4% at Timonette (Artibonite), and 0–1% at the other sites.

Morphological identification provided a first estimate of 25 *R. norvegicus* and 45 *R. rattus* captured. RFLP-PCR confirmed the identification of these two species and corrected misassignment of 6 individuals (especially juveniles or individuals with a part of the tail lost due to injury), for final numbers of 23 *R. norvegicus* and 47 *R. rattus*. In all, 43 females (14 *R. norvegicus* and 29 *R. rattus*) and 27 males (9 *R. norvegicus* and 18 *R. rattus*) were collected. Among these, 13 *R. norvegicus* and 10 *R. rattus* were collected at the end of the rainy season (November 2020), and 10 *R. norvegicus* and 37 *R. rattus* during the rainy season (September 2021). Overall, only *R. norvegicus* (12 of 70 rats) was observed in the urban area of Damien, whereas the rats caught in the rural area of Artibonite were mainly *R. rattus* (47 *versus* 11 *R. norvegicus*).

### Prevalence of *Angiostrongylus cantonensis*

Based on dissection and direct examination of the heart and pulmonary organs, *A. cantonensis* adult worms were found in only 5 rats (1 *R. norvegicus* and 4 *R rattus*), *i.e.* with an overall prevalence of 7.1% (5/70) [CI 2%–16%]. The highest number of worm specimens (*n* = 20) was found in the pulmonary arteries of a single rat, *R. rattus.* The pulmonary arteries of the other three rats contained 3, 2, and 1 worms, respectively. One worm was recovered from the heart of an *R. rattus* individual*.*

*Angiostrongylus cantonensis* DNA was detected in 22 rats (31.4%), a 4.4-fold higher prevalence of the parasite than recorded by the macroscopic approach. Of note, one rat was negative to the molecular test on the lung, but had a worm in the heart, and was therefore considered positive. The overall prevalence of *A. cantonensis* in the rats was consequently 32.9% [CI 22%–45%].

Prevalence of the parasite was 26.1% [10%–48%] and 36.2% [23%–51%] for *R. norvegicus* and *R. rattus*, respectively*.* The prevalence did not differ significantly between species (Chi-squared test, *p*-value = 0.34). Of the 23 *R. norvegicus*, 2 females and 3 males were infected, and these 5 individuals were 1, 6, and 7 months old; juvenile and adult rats of both sexes could be infected by the parasite. Of the 47 *R. rattus*, 14 females and 4 males were infected, ranging in age from 1 to 15 months, with 4-month-old individuals being the most heavily infected (5 of the 7 4-month-old individuals captured were infected).

### Infection/coinfection with other strongylids

Molecular analyses of worms recovered from the heart and lungs and pan-strongylid DNA detection in lungs revealed infection of *R. rattus* individuals by other nematode species. Seven individuals harboured *Nippostrongylus brasiliensis*, including two co-infected with *A. cantonensis*, and another was infected by *Strongyloides venezuelensis.* Infection or co-infection with other nematodes was not observed in *R. norvegicus*.

### Geographical distribution of *Angiostrongylus cantonensis*

Four sites out of 8 were positive for *A. cantonensis*: 3 rural sites in Artibonite and 1 site in Damien (urban area of Port-au-Prince). The site of Timonette (Artibonite) had the highest number of captured rats (43), of which 17 were infected (16 *R. rattus* and 1 *R. norvegicus*). Twelve *R. norvegicus* were trapped in Damien, including 4 infected individuals. Fewer than 5 rats were caught at each of the other sites, with evidence of *A. cantonensis* at two other Artibonite sites (Village Noé and Chatelain).

### Phylogenetic analysis

Seven *A*. *cantonensis* worms provided a sequence reliable for taxonomic comparison. These seven worms were isolated from four rats, all caught in Timonette. The seven sequences were identical. Sequences were deposited in the NCBI database (GenBank numbers: PP178292–PP178298). All belonged to clade II-G, previously identified by Tian *et al.* [41].

**Figure 2:**
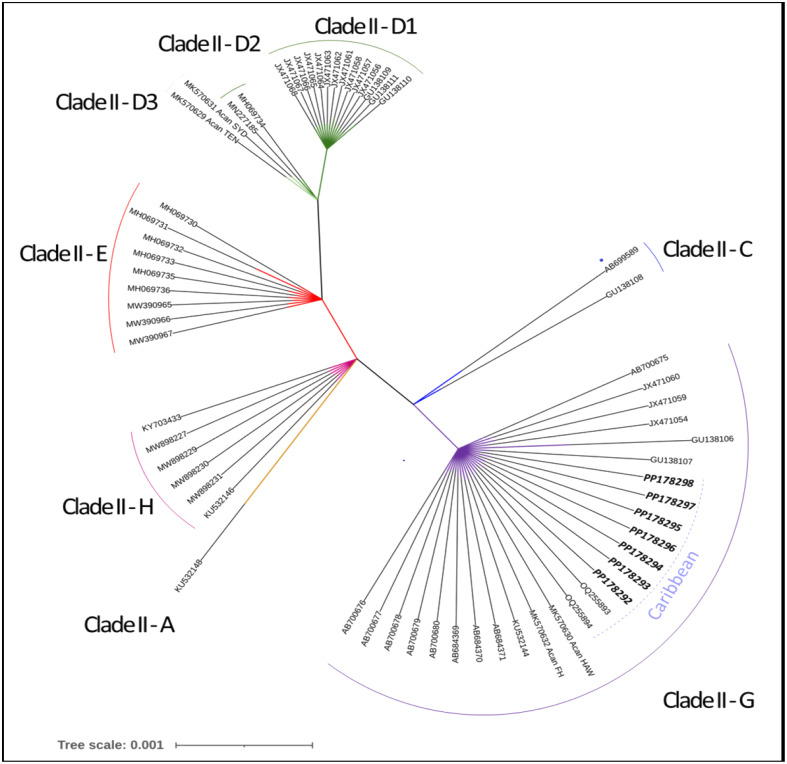
Phylogenetic relationships in Clade II subgroups (*cox1*). The tree was generated using the NGPhylogeny.fr online tool and iTOL visualisation software. Haitian sequences (accession numbers PP178292–PP178298), belonging to Clade II-G, are labelled in bold italics. Other sequences belonging to Clade II-G originated from Japan (AB700675–AB700680; AB684369–AB684371), Thailand (KU532144), French Polynesia (MK570632), Hawaii (MK570630), Guadeloupe (OQ255893–OQ255894), and Brazil (GU138106–GU138107; JX471054–JX471060).

The phylogenetic tree is shown in Figure 2. Of note, sequences of Haitian *A. cantonensis* did not differ from those obtained from Guadeloupe (GenBank OQ255893–OQ255894).

## Discussion

Our study confirms that Haiti is an endemic area for *A. cantonensis.* We observed a prevalence of the parasite in Port-au-Prince suburbs similar to that observed 20 years ago by Raccurt *et al.* [35]. We provided molecular proof of worm identity and highlighted that this parasite was also highly prevalent in Artibonite, therefore encompassing both rural and urban areas.

Infected rats and gastropods were reported from the southern coastal region of the Dominican Republic by Vargas *et al.* [44], who found that all rats examined (*n* = 5, *R. norvegicus*) were infected [44]; thus the island of Hispaniola as a whole should be considered as endemic for *A. cantonensis*, like many of the neighbouring Caribbean islands (Cuba, Jamaica, Puerto Rico, and French Antilles) [1, 6, 9, 10, 46]. Notably, the overall prevalence of *A. cantonensis* in Haitian rats in our study (32.9%) is close to that reported in Jamaica, 32.0%, *n* = 437 (297 *R. rattus* and 140 *R. norvegicus*) [46], where human eosinophilic meningitis is a public health concern.

Although we did not detect any differences in *A. cantonensis* prevalence between the two rat species, we observed infections or co-infections by other strongylids only in *R. rattus.* This may be explained by differences in food preferences and/or host susceptibility [47], and the true impact of coinfections on *A. cantonensis* population dynamics remains to be explored.

Amplification of parasite DNA from lung tissue allowed highly sensitive detection of *A. cantonensis*. Such an approach has previously been used for retrospective rodent surveys using cryopreserved samples [31]. We observed an unexpected four-fold higher prevalence than using conventional macroscopic detection of adult worms in pulmonary arteries and/or the right ventricle. Delayed dissection of lungs and heart stored for one year in ethanol could have led to missing morphologically altered adult worms, compared to dissection of fresh tissues. Also, DNA detection in lung tissue may detect parasite eggs, L1 larvae or residual cells shed from adult worms. Moreover, rats with positive PCR results from lung tissue could have been recently infected, with L3 larvae present in their bloodstream. Qvarnstrom *et al.* showed the higher sensitivity of quantitative PCR, *i.e.* 100% versus 54% detection in 37 rats [34]. Similar comparative studies using optimal preanalytical conditions should be conducted to compare performances of morphological examination versus conventional PCR performed on lung tissues.

Sequencing of *cox1* from seven adult worms detected only a single haplotype of *A. cantonensis* in Haiti. Phylogenetic analysis revealed that the Haitian haplotype belonged to clade II according to Tian *et al.* [41]. According to these authors, Clade II is the overwhelming variant beyond Southeast and East Asia, except for a small number of samples of Clade IV and Clade V in Hawaii and Rio de Janeiro, respectively. Moreover, the Haitian haplotype belonged to Clade II-G, which is the most common type, accounting for almost half of the haplotypes of this clade described so far (Fig. 2). Recently, Gamiette *et al.* described a Clade II-G haplotype of *A. cantonensis* from Guadeloupe, French Antilles, which also belongs to clade II-G [14]. Including our isolates and those reported by Gamiette *et al.*, clade II-G has now been detected in 9 locations worldwide (Japan, French Polynesia, Hawaii, Spain, continental USA, Vietnam, Brazil, Guadeloupe, and Haiti). Given that haplotype diversity of *A. cantonensis* was significantly higher in Southeast and East Asia, and that the New World showed higher diversity of the major clade II in contrast to the Pacific, Tian *et al.* speculated that the rat lungworm originates from Southeast Asia rather than from the Pacific [7, 41]. However, how clade II-G *A. cantonensis* spread to the Caribbean remains to be determined.

Potential intermediate hosts for *A. cantonensis* have not been investigated in Haiti. Infection of *Subulina octona* was previously shown by Vargas *et al.* in the Dominican Republic and by Andersen *et al.* in Puerto Rico, while Waugh *et al.* showed that of their 777 snails and slugs examined, 12.5% harboured third-stage larvae of *A. cantonensis* (*Pleurodonte* spp., *Poteria* spp., *Thelidomus aspera*, *Sagda* spp., and veronicellid slugs) in Jamaica. [1, 44, 46]. The giant African snail, *Lissachatina fulica*, a species of terrestrial snail commonly found infected by the parasite worldwide has not been reported from Haiti, Jamaica or the Dominican Republic. Thus, infected rats from cargo ships may have been more important than introduced infected molluscs for expansion of the geographic range of the parasite to the island of Hispaniola and to Jamaica. The situation may be different in the southern Caribbean, where *L. fulica* has been introduced and is now widespread in the French Antilles [9, 10, 14]. Field investigations remain to be done in Haiti to identify intermediate and paratenic hosts involved in the parasite cycle.

## Conclusion

Our study provides molecular evidence of *A. cantonensis* in rodent samples from two different landscapes of Haiti. The presence of this parasite in Haiti since at least 20 years ago should alert healthcare authorities and practitioners to the possible occurrence of neuroangiostrongylosis. The risk to the population is not negligible: firstly, because human infections may sometimes be due to accidental ingestion of infected snails and slugs, and secondly, even though Haitians generally do not eat raw molluscs or crustaceans and raw vegetables are rarely consumed, the dietary habits of a people can change. Further investigations on the presence of the parasite in different hosts and the incidence of eosinophilic meningitis in humans in various landscapes across the country are needed to better understand the distribution and impact of *A. cantonensis* in Haiti.
